# Efficacy of Blood Pressure reduction of Losartan in selected Thai populations using Home Blood Pressure Monitoring and Office Blood Pressure measurements

**DOI:** 10.7603/s40602-015-0003-y

**Published:** 2015-09-22

**Authors:** Sarana Boonbaichaiyapruck, Wirunsiri Mekwiwatanawong, Kanuengnit Srisala, Montawatt Amnueypol, Prasit Keesukphan

**Affiliations:** 1 Cardiology Unit, Department of Internal Medicine, Faculty of Medicine, Ramathibodi Hospital, Mahidol University, 270 Rama-6 Road, Ratchatewi District, 10400 Bangkok, Thailand; 2 Department of Family Medicine, Faculty of Medicine, Ramathibodi Hospital, Mahidol University, 270 Rama-6 Road, Ratchatewi District, 10400 Bangkok, Thailand

**Keywords:** Home Blood Pressure Monitoring, Angiotensin Receptor Blockade, Losartan, Thailand

## Abstract

**Background:**

Angiotensin Receptor Blockades (ARB) is becoming a first line drug for essential Hypertension for many types of patient. Losartan is the prototype of ARB due to its vast clinical trials. Home Blood pressure monitoring can provide accurate evaluation of certain drug effect on blood pressure with small number of patient samples. Local production of medicine has made the Medicine readily available and could bring about clinical improvement. Our hypothesis was that Thai population with essential hypertension responded quite well to Losartan and Generic Losartan was not inferior to Original- Losartan.

**Objective:**

To evaluate the effectiveness and safety in BP reduction by Losartan in certain Thai population and to compare these parameters between Generic Losartan and Original-Losartan using both office and HBPM method.

**Method:**

After a two-week run-in period when they would learn to use HBPM device and their blood pressure were still recorded to be higher than 140/90 by office BP or 135/85 by HBPM with or without previous medical regimen, 24 patients were randomized to receive either Generic Losartan or Original-Losartan for 6 weeks. Then they would cross over to receive the alternative and were followed again at 6 weeks. HBPM was performed in the morning and in the evening for 5 days, at baseline, and after 6 & 12 weeks. Office BP measurements were obtained at baseline and after 6 & 12 weeks.

**Result:**

One patient in each group dropped out from the study. 22 patients with average age of 54 and averaged office BP 154/88 completed the 12 weeks study. By office BP, SBP was reduced by 27±14.2 at week 6 and 28±15.1 mmHg at week 12. By HBPM, SBP dropped by 17±10.8 at week 6 and by 18±9. at week12. At the end of 12 weeks 68% (15/22) of patients had Office BP <140/90 and 64% (14/22) of patients had HBPM <135/85. There was no significant difference of BP reduction at week 6 between Original-xLosartan and Generic Losartan group. After crossover the BP reduction was maintained in both groups. The percentage of patient whose Office BP <140/90 or HBPM <135/85 were not different among the two Losartan groups. There was no serious adverse side effect.

**Conclusion:**

Using both office BP and HBPM this group of Thai patient with essential hypertension responded well to Losartan and Generic Losartan.

## Introduction

Hypertension is a global medical and public health problem, affecting 26.4% of the worldwide adult population in 2000. It is projected to affect 29.2% of the population by 2025^1^. The prevalence of Hypertension is also on the rise in Thailand^2^. Angiotensin receptor antagonists (ARB) have become an established class of medicine for the treatment of hypertension. Their widespread use is related to their recognized antihypertensive efficacy combined with a placebo-like tolerability profile. Losartan has been in Clinical practice in Thailand for over 10 years but is still not readily available due to its import status and price. Local production of medicine has made many valuable medications more affordable but their quality need to reassured. Office BP was used mostly to evaluate the response to antihypertensive therapy. Lately Home Blood Pressure Monitoring (HBPM) was introduced with the potential to rule out the possibility of white-coat hypertension^3^, to improve patient adherence to therapy and to evaluate response to antihypertensive medication^4^. The method is now cited as an alternative approach to characterize BP levels and to estimate the effect of antihypertensive treatment in clinical trials. A prospective study even suggests that HBPM has a better prognostic accuracy than office BP measurement in treated elderly hypertensive patient^5^. We conducted this study to evaluate the response of BP to commonly used angiotensin receptor antagonist Losartan and to see whether there is any difference among the original foreign made brand name and locally produced one.

## Method

After an enrollment visit (Visit 1 [V1]) in which uncontrolled patients were identified and prior antihypertensive therapy were continued. They were instructed to use HBPM device and to return with the recorded data after 2 weeks (Visit 2 [V2]), patients whose BP remained uncontrolled (average home SBP > 135 mm Hg) were eligible to enter the second phase of the study and received 50 mg of either Original Losartan (Cozaar, Merck Sharp & Dome) or Generic Losartan (Lanzaar, Berlin Pharmaceutical ,Thailand ) for 6 weeks. They would return to clinic (Visit 3 [V3]) where they would hand in the booklets containing their home measurement by the device the week before. Then they were switched to receive the other type of Losartan and to return again after 6 weeks of therapy (Visit 4 [V4]). Blood were drawn at visit 2, 3 & 4 for CBC, Electrolyte, Liver function test and Creatinine. This study was approved by Ethic Committee of Ramathibodi Hospital, Mahidol University.

## Office bp measurements and hbpm

The week before randomization (V2) and the two 6 weeks visit (V3 & V4), patients performed HBPM twice a day for 5 days using a validated electronic device (Digital blood pressure monitor UA 767 plus30/A&D Medical Tokyo JAPAN), according to a standard procedure for which they were trained: after 5 min of rest, three seated measurements in the morning (between 6 and 10 AM) at 1-min intervals, just before taking the study drug; and three seated measurements in the evening (between 6 and 10 PM) at 1-min intervals. Data were recorded by hand for each reading in booklets provided. Data from the booklets were transferred to computer on each visit and blinded to treatment allocation. All measurements performed on the first day of each study period (morning and evening) were considered as part of the patient’s training and excluded from the analysis.

Office BP was measured three times at 1-min intervals in a sitting position after 5 min of rest, either manually with a mercury sphygmomanometer or with a validated electronic device; each physician used the same method throughout the study.

The primary assessment criterion was the difference between the groups in the mean SBP and DBP change of office BP from baseline to the first 6 week and after switching to end of study (12 weeks of treatment). The secondary criteria included:

- change in mean SBP (HBPM) at the two timeframes (6 &12 week of treatment)
- change in mean SBP and diastolic blood pressure (DBP) at trough morning values (HBPM) at the two periods.
- percent of patients with “Controlled BP” by office BP < 140/90 HBPM at the two timeframes.
- percent of patients with “Controlled BP” by HBPM <135/85 at the two timeframes.

## Statistical analysis

This study was conducted with triple blind design and we utilized SPSS version 18 for the statistical analyses. We summarized data using means with standard deviations (SD) and proportions for nonparametric data. Normally distributed data were compared using Student’s t-test and proportions with Mantel–Hanzel Chi-square test stratified according to therapy group. Responder was described as those with Office BP (OMBP) less than 140/90 mmHg and HMBP less than 135/85 mmHg. Blood pressure reduction was compared using repeated measurement ANOVA and 90% confidential interval (p- value <0.10) with non– inferiority (1-tailed) was used for statistically significant. By having multiple measurements of blood pressure (3 times twice a day for 4 days) allowed a calculation of at least 20 patients to be included.

## Results

There were 24 patients enrolled in the study. One in each group dropped out by not showing up for 3rd visit for reason of inconvenience. The average age of the whole groups was 54 years and 50% were male. Majority (86%) had known Hypertension and were being treated as shown in *Table*-*1*. After run in period of 2 weeks, the office blood pressure were higher than the original screening measurement. HBPM were lower than office BP but still at the level of qualification (SBP>135mmHg). The whole group responded well at 6 and 12 weeks of Losartan by both office and HBPM. By office BP, SBP was reduced by 27±14.2 at week 6 and 28±15.1 mmHg at week 12. By HBPM, SBP dropped by 17±10.8 at week 6 and by 18±9. at week12. Mean Office BP dropped from 161/93 to 133/83 at wk 6 and was maintained at 133/81 mmHg at wk 12. Mean HBPM dropped from 146/87 to 130/80 at wk6 and was maintained at 128/80 mmHg at wk12. At the end of 12 weeks, 68% (15/22) of patients had Office BP <140/90 and 64% (14/22) of patients had HBPM <135/85.

At second visit HBPM in average was 8 & 1 mmHg lower than office BP for SBP and DBP respectively. With Treatment at week 6 and week 12 this gap narrowed down. Accordingly, the magnitude of BP reduction was larger by office BP than HBPM. The level of average SBP and DBP during treatment was not that significantly different between office BP and HBPM. The two Losartan groups were similar in term of baseline demographic characteristics and baseline run-in BP measurements. In comparison to Original Losartan, Generic Losartan reduced blood pressure to the same degree as shown in Table-2. Both Office BP and HBPM were reduced to a comparable level with similar magnitude of reduction at both week 6 and after switching at week 12. There was no statistical difference on the level of BP achieved and on the magnitude of BP reduction between the two groups using both methods. There were more number of patient whose BP was under controlled by Office BP<140/90 in the Generic-Losartan group at week 6 (81% vs 54%) compared to Original-Losartan group but the difference was not statistically significant (p=0.181). The different was no longer observed at week 12 after switching. Using <135/85 by HBPM as cutoff for “controlled BP” the number of patients were not different among the two groups at both timeframe of measurements. The diurnal fluctuations of BP was not observed using HBPM during the run-in period. There was a trend, however, of fluctuations being observed during active treatment. All Blood tests were within normal limits throughout the study period.

## Discussion

We observed 3 main findings from this study. Firstly, HBPM could be used by most patients in the study to confirm the high BP level observed in the clinic. Secondly Losartan, the prototype of ARB, was efficient in reducing BP in this selected group of Thai population. Thirdly, Generic Losartan performed as well as original made one.

HBPM has been used quite extensively as the mode of confirmation of elevated BP. In Europe, there is increasing awareness of the use of HBPM and the new European guidelines recommended HBPM for the management of hypertensive patients from the diagnosis to the followup^6^. JNC-7^7^ from the United States and NICE guideline^8^ also supported such a practice. However, there was a limited data of study of such practice in Thailand despite readily availability of the device. Our research nurse spent sometime instructing our patient to use the device before embarking the study. They were all comfortable in using it. By reducing the variability of blood pressure estimates, self measurement improves the sensitivity of clinical trials and makes it possible to reduce substantially the sample size of patients in the trials. It has the potential to detect even minor blood pressure changes^9^. The fact that we used manual recording of BP instead of device memory because we feared that the device could be used in other people such as relatives of friends. One study from Thailand supported the use HBPM as a mode of evaluation from behavioral therapy on BP before any adjustment should be made in the medical regimen^10^. Moreover, measurements performed in the morning before drug intake allows the evaluation of the residual efficacy of antihypertensive treatment^11^. By having 3 measurements twice a day for 4 days of blood pressure made the numbers more reliable thus allow a relative small number of patients in our study.

ARB has been in clinical practice for more than a decade and Losartan has been one the of the popular ARB^12^. The reason of their popularity were its ease of use and extensive clinical studies. Compare with other antihypertensive agent, ARB was retained at 1 year more than any other medicine. None of our patients reported any side effect though it is a short time interval. All blood tests of CBC, Electrolyte, Liver and kidney function tests did not change in the study period. LIFE study^13^ was the first one to suggest superiority of ARB to Betablocker and using Office BP as the measurement of BP response in addition to clinical endpoints. Sitting systolic blood pressure at end of follow-up or at last visit before a primary endpoint occurred, if one did, fell by 30.2 (18.5) and 29.1 mm Hg (19·2) in Losartan and Atenolol groups, respectively (treatment difference p=0.017). Mean blood pressures at last visit were 144.1/81.3 (17.1/9.6) and 145.4/80.9 mm Hg (16.4/9.6) respectively, in Losartan and Atenolol groups. Blood pressure of less than or equal to 140/90 mm Hg was achieved in 2268 (49%) and 2099 (46%) Losartan and Atenolol patients, respectively. The study was carried out in those hypertensive who also had left ventricular hypertrophy from EKG. The composite endpoints of stroke, myocardial infarction and heart failure were significantly lower in Losartan than in Atenolol group. LIFE study and other confirmed the benefit in Diabetic and the elderly with isolated systolic hypertension^14^. The recent expiration of Losartan patent prompt local production of the drug. There was a need to evaluate the response of Losartan in our population. Searching the database we came up short on study of Losartan in Thai population in term of BP reduction efficiency. This study was conducted to see the BP reduction of Losartan in Thai population and to evaluate such efficiency of the local made version rather than bioavailability aspect.

Majority of the population we selected were those with known hypertension who were being treated with medications but ARB was not in the regimen. Minority were new cases of hypertension. All of them agreed well with the medication and the drop out was not from the side effect from the drug. There was no drug interaction and no abnormality of Electrolyte, Liver function or Creatinine were observed. It did confirm Losartan ease of use with Placebo-like side effective of Losartan in this group of Thai population. Losartan lower blood pressure effectively with office BP, SBP reduced by 27±14.2 at week 6 and 28±15.1 mmHg at week 12. Using HBPM, SBP dropped by 17±10.8 at week 6 and by 18±9. at week12. These parameters were quite comparable to the original study of Losartan. 68% & 64% of patients became controlled by having thier BP in the target range.

The two types of Losartan i.e. Generic Losartan and Original Losartan were equally effective in lowering BP by both office BP and HBPM. Percentage(%) of patients whose BP were in the “control range” were also similar for both agents. This has an implication that cheaper locally made Losartan can be prescribed more widely with expected similar control of one of the significant risk factors for heart disease and stroke. Losartan is being manufactured by many local pharmaceutical company in Thailand and ASEAN country. The ASEAN community is soon to be in operation and the medicine with good study support should provide confident to the public and the physician. This would include the efficiency and safety for which this study of Generic Losartan suggested.

**Figure-1:**
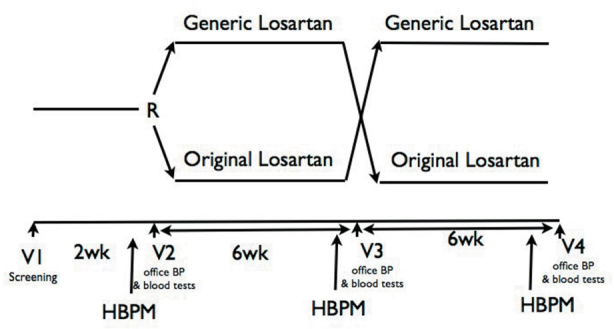
Protocol of the study

## Conclusions

Home Blood Pressure Monitoring (HBPM) were used to confirm the uncontrolled BP level in a number of Hypertensive Thai patients and it was used to evaluate the efficiency of treatment by Angiotensive Receptor Blockade, Losartan. Losartan effectively lowered BP by both office BP and HBPM method with magnitude of BP reduction in the expected range.

## Acknowledgements

Department of Family Medicine, Outpatient Department and Personnel of Cardiology Unit, Department of Internal Medicine, Ramathibodi Hospital, Mahidol University.

What does this study add to the already known science in this field: The efficacy of Losartan in lowering blood pressure is already well known and Home Blood Pressure Monitoring is becoming more accepted as mode of evaluation. This study adds the dose response of such drug in Thai population using Home Blood Pressure Monitoring as the indicator. It also adds that Generic Losartan performed as well as Original Losartan in achieving blood pressure control.

**Table-1:** Baseline characteristics and clinical parameters comparison

Clinical parameters	All (N=22)	Group A (N=11)	Group B (N=11)	P - value
Age (years)	54±15.57	52±17.64	57±13.47	0.4245
Weight (kg.)	73±18.30	76±13.58	70±22.4	0.4850
Height (cm.)	163±8.72	163±9.49	163±8.34	0.9812
Male	11	5	6	0.6700
Treated HTN	19	9	10	0.5310
Alcohol	8	4	4	0.368
Caffeine	16	8	8	0.513
Smoking	0	0	0	-

**Figure-2:**
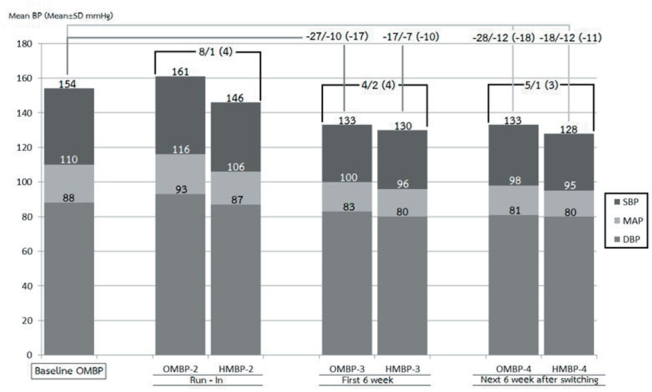
Office and Home Monitoring Blood Pressure response of the whole group to Losartan at Run-in period, at week 6 and week 12

**Table-2:** Magnitude of BP reduction during the treatment. Both type of Losartan lower BP to a similar extent and Generic is not inferior to Original Losartan

	Group A		Group B	
	Office BP	HMBP	Office BP	HMBP
**First 6wk**	**Original Losartan**		**Generic Losartan**	
Systolic	-24	-12.55	-30.91	-20.73
Diastolic	-6.91	-5.45	-13.73	-8.27
Mean	-13.64	-7.74	-19.45	-12.3
**Second6wk**	**Generic Losartan**		**Original Losartan**	
Systolic	-27.64	-15.36	-28.27	-21.18
Diastolic	-9.36	-7.45	-14.45	-7.27
Mean	-16.73	-9.99	-19.09	-11.79

**Figure-3:**
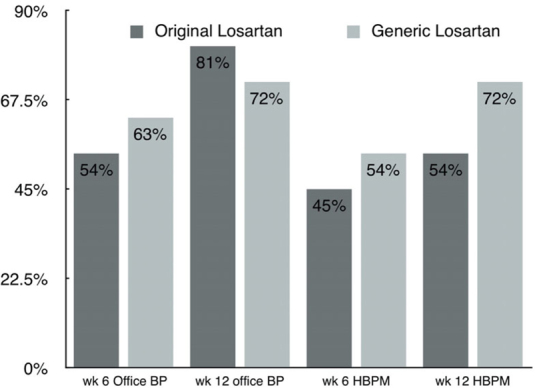
Percentage of ‘Controlled BP’ by Office BP <140/90 and by HBPM <135/85 at 6 and 12 week of treatment.

**Table3:** Result of BP changes during run-in; after 6 weeks & then after switching for another 6 weeks. All parameters were comparable between Original Losartan & Generic Losartan

		Group A(N = 11)		Group B(N = 11)	
**Initial qualify**	Systolic	159±10.3		163±8.6	
	Diastolic	92±10.7		93±12.1	
	Mean	116±5.6		117 ±8.8	
		**Office BP**	**HMBP**	**Office BP**	**HMBP**
**Run-in**	Systolic	151 ±8.75	146±10.25	157±9.86	147±9.21
	Diastolic	88±10.61	90±8.76	88±13.1	84±8.37
	Mean	109±7.35	108±7.71	111 ±10.12	104±6.75
		**Original Losartan**		**Generic Losartan**	
**First 6 week**	Systolic	135±5.97	133±9.41	132±16.5	126±9.3
	Diastolic	85 ±8.44	85±9.81	80±11.25	76±8.48
	Mean	102±6.25	100±8.53	97±11.31	92±8.11
		**Generic Losartan**		**Original Losartan**	
**Second 12 week**	Systolic	131 ±10.55	131 ±7.39	135±17.71	126±9.75
	Diastolic	83 ±9.48	83±8.1	79±13.63	77±9.71
	Mean	99±8.32	98±7.14	98±14.14	92±8.82
